# The Merits, Limitations, and Future Directions of Cost-Effectiveness Analysis in Cardiac MRI with a Focus on Coronary Artery Disease: A Literature Review

**DOI:** 10.3390/jcdd9100357

**Published:** 2022-10-17

**Authors:** Taha A. Siddiqui, Kiran S. Chamarti, Leila C. Tou, Gregory A. Demirjian, Sarah Noorani, Sydney Zink, Muhammad Umair

**Affiliations:** 1 Philadelphia College of Osteopathic Medicine, 625 Old Peachtree Rd. NW, Suwanee, GA 30024, USA; 2 Charles E. Schmidt College of Medicine at Florida Atlantic University, 777 Glades Road BC-71, Boca Raton, FL 33431, USA; 3 Feinberg School of Medicine, Northwestern University, 420 E Superior St., Chicago, IL 60611, USA; 4 Department of Computer Science, Northwestern University, 633 Clark St., Evanston, IL 60208, USA; 5 The Russell H. Morgan Department of Radiology and Radiological Science, The Johns Hopkins Hospital, 601 N Caroline St., Baltimore, MD 21205, USA

**Keywords:** cost-effectiveness analysis, CMR, cardiac magnetic resonance imaging (cardiac MRI), coronary artery disease, quality-adjusted life year (QALY), literature review

## Abstract

Cardiac magnetic resonance (CMR) imaging has a wide range of clinical applications with a high degree of accuracy for many myocardial pathologies. Recent literature has shown great utility of CMR in diagnosing many diseases, often changing the course of treatment. Despite this, it is often underutilized possibly due to perceived costs, limiting patient factors and comfort, and longer examination periods compared to other imaging modalities. In this regard, we conducted a literature review using keywords “Cost-Effectiveness” and “Cardiac MRI” and selected articles from the PubMed MEDLINE database that met our inclusion and exclusion criteria to examine the cost-effectiveness of CMR. Our search result yielded 17 articles included in our review. We found that CMR can be cost-effective in quality-adjusted life years (QALYs) in select patient populations with various cardiac pathologies. Specifically, the use of CMR in coronary artery disease (CAD) patients with a pretest probability below a certain threshold may be more cost-effective compared to patients with a higher pretest probability, although its use can be limited based on geographic location, professional society guidelines, and differing reimbursement patterns. In addition, a stepwise combination of different imaging modalities, with conjunction of AHA/ACC guidelines can further enhance the cost-effectiveness of CMR.

## 1. Introduction

The utilization of cardiac magnetic resonance (CMR) in cardiac imaging became possible with fast acquisition techniques and improved imaging quality, allowing adequate assessment of cardiac function and morphology with a high degree of accuracy and precision [1,2]. CMR is hailed as the single most important procedure that could revolutionize the standard of care [3]. However, given the relatively long duration of CMR examination, its costs, and multiple limiting patient factors including patient comfort and magnetic resonance imaging (MRI) safety, other diagnostic alternatives with lower cost and shorter turn-around times are typically preferred to attain preliminary information [2,4,5].

CMR can be employed to evaluate the anatomy and function of the cardiac chambers, multiple valvular pathologies, scarred myocardium, and tissue characterizations [2,6]. It is the preferred imaging modality to differentiate patients with ischemic and non-ischemic cardiomyopathy, particularly in patients diagnosed with myocarditis [2,7]. T1 and T2 parametric maps are especially useful in tissue characterization regarding inflammation, which assists in diagnosis, predicting prognosis, and clinical decision-making for myocarditis patients [7]. The ability of CMR to evaluate myocardial scar patterns, examine myocardial wall thickness in hypertrophic cardiomyopathy, and measure ejection fraction in patients with heart failure makes it a valuable tool when assessing patients with defibrillators or resynchronization therapy [8]. While echocardiography (ECHO) also provides some information in these conditions, CMR is preferred due to its superior accuracy in assessing ventricular volume and ejection fraction, because it has improved endocardial definition, the capacity to create 3D images, and clear delineation of blood-myocardial boundaries [8,9]. In addition, valvular and congenital heart diseases are often diagnosed using ECHO, but the use of CMR can serve as diagnostic confirmation and assist in prognostic evaluation [9]. CMR is also considered the most accurate imaging modality for the assessment of vascular distribution and myocardial scarring to evaluate tissue viability following infarction [3]. Interpretation of the degree of scarring with the use of delayed gadolinium enhancement can help physicians gauge the chance of tissue recovery in the event of tissue ischemia followed by reperfusion [2]. In cases where there is a question of inducible ischemia, a simultaneous stress perfusion MRI, for which pharmacological agents may be administered to simulate myocardial stress physiology and identify inducible ischemia, may be performed [5]. Microvascular obstruction as determined by CMR has been associated with more frequent cardiovascular complications while infarct size is an important marker for long-term prognosis in patients with acute myocardial infarction [10]. Many myocardial processes that involve microscopic or non-vascular macroscopic fibrosis can readily be detected by CMR as compared to ECHO, which has limited utility in evaluating scar tissue [11,12]. CMR is also the modality of choice in assessments of many non-ischemic pathologies including non-ischemic cardiomyopathies, myocarditis, myocardial tumors, pericardial diseases, cardiotoxicity, inflammatory conditions, valve abnormalities, infiltrative conditions, cardiac masses, and other genetic, metabolic, or idiopathic cardiomyopathies [11,12,13,14,15].

Despite its role as a key imaging technique in cardiac phenotyping, there are several areas for improvement in the utilization of CMR. One limitation is its time-consuming nature, especially in patients with irregular heart rates and rhythms that disrupt the image quality [4]. Besides patient-related factors, the examination time and post-processing time for CMR further depend on pulse sequences, 2D or 3D acquisition, reader experience, and analysis methods (i.e., software platforms, mathematical models, contour detection method) [8]. In addition, breath-holding for 10 to 20 s is essential for obtaining high-quality images to reduce artifacts [16]. Patient-related factors or intolerance to the examination can result in decreased quality of the images. Patients who are acutely ill and unable to hold their breath may instead undergo an ultrafast, real-time version of delayed contrast-enhanced MRI, but this technique has a slightly reduced sensitivity and may underestimate the transmural extent of an infarction [16]. In addition, there are several new techniques, such as compressed sensing, available to reduce the scan time in such cases [17]. CMR may be logistically disadvantageous when compared to other imaging techniques. For example, ECHO equipment is portable while CMR requires the patient to relocate to the scanner [9]. Due to limitations imposed by patient-related, institutional, and logistic factors, CMR is often under-utilized, despite being a standard modality of choice for the evaluation of numerous cardiac pathologies.

The cost of CMR is an important factor for its appropriate utilization and the just allocation of healthcare resources due to cost being a barrier to entry in community settings. A study done in 2005 found that when compared to ECHO, the costs of computed tomography (CT), single-photon emission computed tomography (SPECT), CMR, positron emission tomography (PET), and right/left heart catheterization were 3.1, 3.3, 5.5, 14.0, and 20.0 times greater, respectively [18]. The decision of the optimal imaging modality is highly variable depending on national (i.e., healthcare system), institutional (i.e., insurance reimbursement), and individual (i.e., disease pre-test probability) factors. For example, a 2017 study in Brazil found that CMR was not as cost-effective when compared to other modalities, largely due to the lack of reimbursement from the national health system which only reimbursed electrocardiogram (ECG), stress ECHO, and SPECT [19]. In this regard, we conducted a literature review on the cost-effectiveness analysis of CMR in determining the diagnosis and prognosis of various cardiac pathologies.

## 2. Materials and Methods

**Figure 1:**
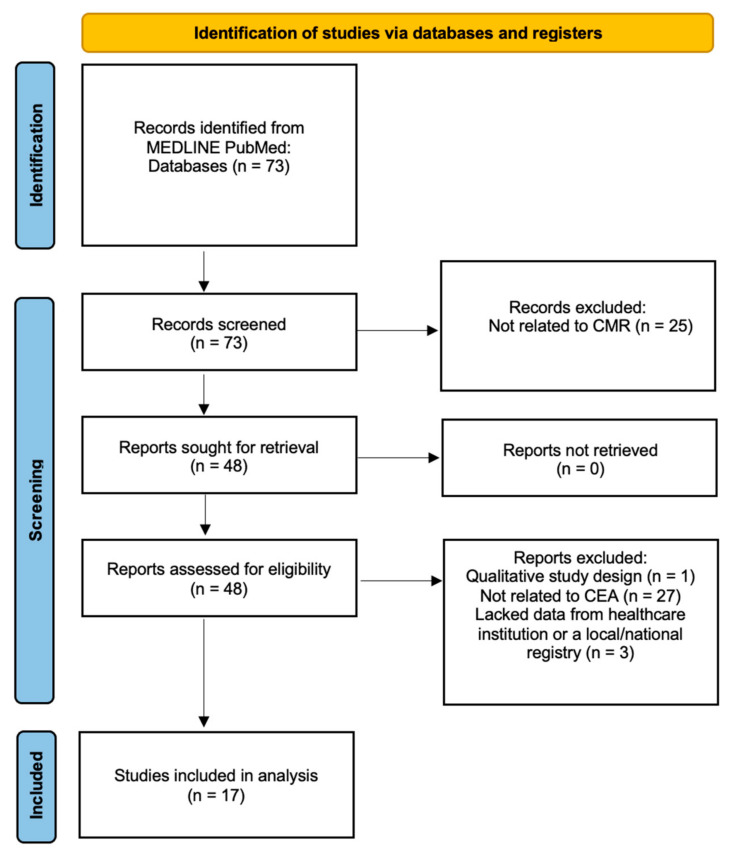
Flow diagram summarizing the identification of included studies (Abbreviations: CEA—Cost-Effectiveness Analysis. CMR—Cardiac Magnetic Resonance).

Articles were searched on the PubMed database using keywords including “cost-effectiveness” and “cardiac MRI” to yield a total of 73 search results (Figure 1). Selected articles were required to include their data from a registry or healthcare institution for which cost analysis was performed to show the cost-effectiveness or lack thereof in the utilization of CMR. Studies included both sexes, all age groups, and all cardiac pathologies. Qualitative descriptive studies on the use of CMR and studies in a language other than English were excluded. Articles with redundant information and overlapping patient cohorts were not included in this review. We screened by abstracts and methods of the articles to find relevant studies and selected only those that had sufficient cost-analysis data. Eventually, 17 studies that provided the most relevant information were selected for this review (Table 1).

## 3. Cost Analysis Models

With the growing use and cost of CMR and other cardiac imaging modalities, governments, insurance companies, and other stakeholders are interested in the financial impact of these examinations, especially with a focus on the cost-effectiveness of such interventions [36,37]. Because the cost-effectiveness of a diagnostic modality depends on factors more than simply the cost of the procedure (e.g., fee for the technician, interpreter, electrical costs) that can vary from one health institution to another, it is often difficult to ascertain the true cost of a procedure [36]. To address such concerns, cost analysis models can be employed for a particular imaging modality, with cost-effectiveness analysis being preferred in medical and cardiovascular imaging.

Imaging modalities can be analyzed using four different cost analysis models, which are cost–benefit, cost-effectiveness, cost-minimization, and cost-utility. Although these three approaches seem interchangeable, there are distinct differences between them. Cost–benefit analysis, in which both the cost and benefit are measured in terms of currency through monetizing health and duration of life, is problematic as placing a monetary value on human lifespan can be ethically conflicting and subjective based on different viewpoints [20,36]. Cost-utility is appropriate to use when cost savings have been demonstrated between the imaging modality and its alternatives, and when obvious imaging preference or utility data is available [36]. The lack of this data can make cost utility an inappropriate choice for cost analysis. In contrast, the use of cost-effectiveness analysis allows for the determination of a strong association between human health and the results of an intervention, such as enhanced annual survival or number of years without disease, without monetizing survival and duration of life [21]. Specifically, quality-adjusted life years (QALY) is a measure often used to investigate economic evaluation in cost-effectiveness analyses. This metric captures survival and health-adjusted quality of life; it is calculated by multiplying the quality of life and the quantity of life to measure improvements in morbidity and mortality when comparing two treatment groups by taking the difference in their respective QALYs [38]. The derivation of the cost-effectiveness ratio accounts for all the resources utilized in a particular procedure (i.e., direct and indirect cost-of-care, including physician and technician costs, electricity, hospital fee, loss of work, etc.), and can standardize true procedural costs throughout a healthcare system in a particular population, without creating the ethical issues associated with cost–benefit analysis [36]. Despite these advantages, there are several limitations to the use of this model. One of these limitations is that these models are only applicable to the population from which the data is originally generated [22]. For example, the cost-effectiveness analysis of CMR in patients with differing severity of CAD cannot be used for a broader population without CAD [36]. Because the effectiveness of an imaging modality can drive the outcomes of a treatment, cost-effectiveness analysis must account for these downstream consequences. For example, one study demonstrated the clinical impact of CMR on the treatment plan in five patients who had a change in diagnosis from idiopathic congestive heart failure to noncompaction and spiral hypertrophic cardiomyopathy, leading to major changes from their initial treatment plan [13]. Another instance where imaging drives the treatment is the case of CAD. When a nuclear cardiology stress examination or stress CMR is performed for perfusion abnormalities in the heart, ischemia versus infarct must be determined as revascularization would relieve ischemia but not the infarct [14]. Additionally, CMR without stress perfusion provides valuable information on the viability of infarcted myocardium in the setting of vascular myocardial scar. However, stress CMR also has its utility to identify inducible ischemia as described above. CMR diagnosis also helps with the appropriate management of a patient to optimize the timing of treatment to prevent further complications. Accurate and rapid CMR scans in cancer patients and survivors who have received cancer therapeutics could be beneficial as cancer therapies could lead to cardiomyopathies, myocarditis, or other cardiotoxic effects and heart failure [15]. A rapid CMR scan could lead to early detection of toxicity and prevention of these treatment complications [15]. Similarly, CMR has become an invaluable tool for the early detection of transplant rejection among patients with cardiac transplants [39].

A physician’s clinical judgment to employ specific imaging modalities influences management, treatment plans, and consequent downstream costs. Often, the initial cost of diagnostic testing can be impacted due to this variation [36]. Cost minimization is another cost analysis method that is utilized when multiple modalities with equal clinical significance exist and the cheaper alternative is used [23,36]. Due to the difficulty in quantifying the cost of CMR with other modes of cardiac imaging as they pertain to clinical equivalence, cost-minimization is difficult to use to find a cheaper alternative from an economic standpoint [36]. However, the use of the cost-effectiveness analytical model is greatly applicable in understanding the true costs of CMR.

## 4. Cost-Effectiveness of Cardiac MRI

With the increasing prevalence of many cardiac pathologies, there has been a growth in strategic cardiovascular imaging and a change in the management of these conditions. However, the incidence and prevalence of these cardiac pathologies may be affected by the evolving technology and strategies over the past few decades. A cross-sectional study from 1993 to 2001 showed a three-fold increase in imaging stress tests leading to increased revascularizations and decreased mortality for CAD [21].

The cost and use of cardiac imaging studies (i.e., CMR, CT angiography, SPECT, cardiac PET-CT) vary significantly based on several factors including imaging protocols used in each individual case. The results of these tests can directly change patient management and thus indirectly alter long-term outcomes [20]. The advantages of using certain imaging modalities are not just limited to its direct performance and image quality, but also extrinsic variables including patient demographics, regional considerations, disease prevalence, indication, patient-related factors, logistics, scheduling, duration of the examination, alternative modalities available, and implications of the results [20]. Each imaging modality has its individualized advantages, but studies have shown that a combination of different modalities with very specific indications can sometimes be a better diagnostic strategy in certain circumstances based on overall long-term cost-effectiveness [20].

Cost-effectiveness analysis can vary considerably with patient demographics. For example, regional differences in the incidence and prevalence of pathology also affect the cost-effectiveness of imaging modalities [20]. It is difficult to conduct cost-effectiveness analyses in the United States compared to European countries that utilize universal healthcare models because the cost-to-charge ratios vary considerably among different healthcare institutions and third-party payers [20]. Specifically, the “charge” is the amount billed to the consumer, and often the real cost is a small fraction of the charged amount, which varies from one state to another and amongst different hospitals due to private billing policies [20]. Most often, the price charged for a particular CMR examination varies from the reimbursements by the insurance company due to pre-determined insurance-hospital contracts and agreements [20,36]. The actual price charged is not a discrete amount, rather it is a culmination of technical services (i.e., use of the technical equipment, technologists’ expertise, etc.) and professional interpretation in addition to the use of contrast or any other periprocedural services [36]. This charged price can also vary from state to state and from one hospital to another, thus the billing code of the United States Center of Medicare and Medicaid Services (CMS) average payments are often used as an anchor for comparative analyses [36].

### 4.1. Cost-Effectiveness in Combination with Other Imaging Modalities

Regardless of such challenges, several studies have explored the cost-effectiveness and clinical utility of CMR. One such study conducted a cost–benefit analysis on a modeled population of patients with acute myocardial infarction (MI) to compare the cost of standard therapy (dual antiplatelet therapy and/or aspirin for life) versus CMR-guided management over a 10-year period [24]. Researchers used a hypothetical population consisting of 2000 patients with acute MI and normal coronary angiography [24]. Half of the patients were assigned to standard therapy and the other half to CMR-guided management. Analysis was performed based on United Kingdom costs with outcomes varying based on the time period [24]. Within the first year, the routine use of CMR to identify patients with a true MI increased spending by 14% per patient. By seven years, CMR-guided practice was cost-neutral. After 10 years, CMR-guided management was found to reduce costs by 3% per patient [24]. These findings suggested that while CMR-guided management of acute MI may have increased costs in the first few years, it is cost-effective when managing long-term patients. Another similar study that collected data on a cohort of cardiology referrals in a European multicenter registry concluded that over two-thirds of the 11,040 patients (with indications of myocarditis/cardiomyopathies (32%), suspected CAD (31%), myocardial viability assessment (15%)) experienced appropriate changes in therapeutic management based on CMR results, and in a minority of patients (16%), an entirely new diagnosis was discovered [20]. For instance, the study described a patient who presented to the emergency department with heart failure symptoms and was diagnosed with severe aortic stenosis with a valve replacement recommendation [20]. The patient was later found to have amyloidosis, as evidenced by subendocardial enhancement, with mild aortic stenosis on CMR evaluation [20]. Before undergoing CMR, most patients (64.1%) in the registry had undergone transthoracic ECHO with the remaining cohort undergoing coronary angiography (25.1%), cardiac CT (1.8%), and SPECT (0.8%), prompting further clinical evaluation with CMR due to uncertainty of prior imaging results. Despite these findings, the applicability of these results in North American and Asian countries remains relatively unknown, due to major differences in healthcare systems, cultures, disease prevalence, and social status which predispose to challenges in interpreting the transference of economic effectiveness [20].

### 4.2. The Role of Diagnostic Accuracy

The diagnostic accuracy of CMR for cardiac diseases drives its cost-effectiveness. One study aimed to identify key determinants for the cost-effectiveness of CMR in the United Kingdom in patients with multivessel CAD and unobstructive coronary arteries [25]. The patients were divided into two models of multivessel CAD and unobstructed coronary arteries using an index angiogram and underwent initial percutaneous coronary intervention. The group with multivessel CAD underwent one of three diagnostic and treatment pathways: CMR, fractional free flow (FFR), or stress ECHO. Although there was a small QALYs increase for CMR and FFR compared to stress ECHO, with an increased prevalence of ischemia, it was predicted that FFR would have reduced costs due to simultaneous revascularization during ischemia testing [25]. The reasoning for this prediction was the low ischemia rate of 35% in the FFR group where most patients did not require additional testing or treatment [25]. Despite FFR and CMR having the same QALY increase in this model, CMR was less costly than FFR (£5431 vs. £5855) in terms of overall costs over 1 year. The second model for unobstructed coronary arteries had two diagnostic pathways which were CMR and stress ECHO, or stress ECHO alone. Through the second model, the addition of CMR resulted in a decrease in costs due to treating fewer patients with MI, but this decrease in cost only partly compensated for the additional cost of CMR [25]. However, the two models indicated that the sensitivities and specificities of the imaging modality played a significant role in determining the cost-effectiveness of the overall strategy.

### 4.3. Cost-Effectiveness of CMR in Conjunction with Guideline Recommendations

The use of CMR has also shown to be cost-effective when used in conjunction with guidelines from different cardiology organizations including the American Heart Association (AHA) and the American College of Cardiology (ACC), as well as radiology committees such as the Society for Cardiovascular Magnetic Resonance (SCMR) [40,41,42]. A retrospective study of 361 patients in the United States compared CMR with other imaging modalities [13]. The study had 350 patients who met the AHA and ACC criteria for CMR imaging based on having a limited ECHO (27%), valvular disease (26%), cardiomyopathy (20%), tissue viability (16%), and aortic or vascular disease (11%). There were 11 cases of inappropriately ordered CMR, including eight for left ventricular hypertrophy and three with inadequate justification [13]. Among the 361 patients, 353 had conclusive results based on the CMR findings, and there was a major change in the plan of care for 256 patients. In this study, CMR had an overall net healthcare saving of $833,037 and a per-patient cost saving of $2308. The study concluded that CMR was cost-effective when used in conjunction with the AHA and ACC criteria [13].

Furthermore, a cohort study of 1158 patients from 1 January 2003, to 31 December 2004, in Germany examined the utilization of CMR in stable CAD patients [26]. The inclusion criteria required a clinical presentation of stable CAD with controls for demographic parameters such as age, gender, and cardiovascular risk factors. The exclusion criteria included prior cardiac transplant, left ventricular ejection fraction ≤40%, and known CAD based on angiography. Eventually, of the 1158 patients, 502 patients enrolled in the study with 209 and 293 patients allocated to the CMR and coronary angiogram groups, respectively. Direct CMR was used in accordance with the SCMR recommendations. Due to coronary angiography being a morphological modality and CMR being a functional study (e.g., exercise-induced abnormalities), different rates of CAD diagnoses were measured in the two groups with CMR having the lower prevalence [26]. Patients undergoing CMR had a savings of 12,466€ in hospital costs per life year. These savings could be associated with the CMR group managed in a mainly ambulatory setting compared to the coronary angiography group managed in an inpatient setting. The CMR group also had shorter hospital stays and decreased coronary angiogram interventions, leading to overall savings [26].

Similar results were also found in the prospective study known as Stress Cardiac Magnetic Resonance Versus Computed Tomography Coronary Angiography for the Management of Symptomatic Revascularized Patients (STRATEGY). This study compared coronary CT angiography (CCTA) with CMR in terms of overall cumulative costs over one year and cost-effectiveness of the index examination in 600 symptomatic CAD patients with a history of revascularization. In this study, the CMR group not only had lower CAD spending and downstream costs using both invasive and noninvasive imaging but also had lower radiation exposure compared to the CCTA group [27]. Over the course of one year, the cumulative costs (with cost of index test included) for the CCTA group averaged 2012 ± 2888€ while the CMR group averaged 1516 ± 2464€. A comparison of costs for each examination revealed that CCTA (218 ± 298 €/y) had a higher cost-effective ratio compared to CMR (119 ± 250 €/y) [27]. One of the explanations for the higher cumulative costs in the CCTA group is the higher rate of additional noninvasive imaging and invasive coronary angiography when abnormal findings arise. With increased invasive coronary angiography, there were increased stent interventions due to the oculostenotic reflex, which is the reflexive revascularization upon visualizing stenotic coronary vasculature even if the vessel is unlikely to cause problems in the future [27,43]. This study highlights the effect of disease progression and treatment history on selecting the appropriate imaging modality; it is more cost-effective for patients with known CAD and a history of revascularization to undergo CMR instead of CCTA as this is more likely to avoid unnecessary revascularization. In terms of major cardiac adverse events, the CMR group had a lower rate of 5% compared to the 10% in the CCTA group. Taking these three studies together, guidelines used in conjunction with CMR demonstrated overall net savings and cost-effectiveness compared to other imaging modalities.

### 4.4. Stepwise Testing with Other Imaging Modalities

Imaging strategies with stepwise testing may result in higher cost-effectiveness and increased QALYs compared to a single imaging modality alone or starting with the most definitive examination [28,29]. One study conducted in the United Kingdom investigated eight different diagnostic strategies with combinations of exercise tolerance test (ETT), CMR, SPECT, and coronary angiography in a patient population from the CE-MARC study that was referred to cardiologists with suspicion of angina pectoris [29]. In the study population, a pre-test probability of 40% (requiring revascularization due to significant stenosis) was used for cost-effectiveness analysis (outcomes measured in QALYs), with 15.9% of the patients suspected to have CAD without significant stenosis. With the lower cost-effective threshold of £20,000, the best strategy was the exercise stress test followed by CMR, then coronary angiography. The sequence of tests only proceeded if the prior test was positive or inconclusive. With a higher cost-effectiveness threshold of £30,000, the best strategy was CMR followed by coronary angiography [29]. Both strategies utilized CMR, with the study concluding that the use of CMR is likely a component of the most cost-effective strategy, especially in patients with CAD when the incremental cost of CMR compared with SPECT is not too large (cost increment < £90 at a threshold of £20,000 per QALY, and <£115 at a threshold of £30,000 per QALY) [29]. In contrast, in the strategies that used SPECT followed by coronary angiography, or the use of ETT followed by coronary angiography, CMR had lower cost-effectiveness at both £20,000 and £30,000 per QALY.

### 4.5. Regional Applicability of CMR

Another investigation produced similar results when utilizing evidence from the CE-MARC study and applying it to the Australian healthcare system [30]. This study also investigated eight potential clinical strategies using different combinations of electrocardiogram stress testing (EST), SPECT, stress CMR, and coronary angiography by using a decision analytical model coupled with three distinct Markov models [30]. Based on a cost-effectiveness threshold of $45,000 to $75,000 per QALY gained, the most cost-effective strategy was initial EST, followed by stress CMR if EST was positive or inconclusive, followed by coronary angiography if the stress CMR was positive or inconclusive [30]. A similar trial was conducted in Switzerland with the same eight strategies [28]. However, the conclusion differed as the most cost-effective strategy was ETT followed by CMR, then coronary angiography if CMR was positive or inconclusive. The limitation of the study was that the cost difference between coronary angiography and CMR is smaller in Switzerland compared to the United Kingdom [28]. With different costs for imaging studies including CMR and coronary angiography among various thresholds per QALY, a single cost-effective strategy for moderate to high pretest probability groups may not be uniformly used.

One study compared the use of CMR with invasive coronary angiography from the European registry data in Germany, the United Kingdom, Switzerland, and the United States healthcare systems [31]. The study recruited 2717 patients for the evaluation of a diagnostic workup for CAD. In their study, a cost analysis was conducted on a multicenter European registry where CMR was used as an initial diagnostic modality to assess for myocardial ischemia, with CAD-positive patients being referred to coronary angiography. This was compared to another hypothetical strategy that utilized coronary angiography as a single diagnostic test. By correcting for different healthcare models with outpatient/inpatient procedure coding in these countries, the study determined the cost-reduction of using CMR in a non-emergency setting as compared to invasive coronary angiography. Specifically, when comparing the use of coronary angiography (inpatient) in all these countries, the CMR strategy was associated with an average of 53.5% lower costs. In addition, all tests (coronary angiography, SPECT, CT, CMR, ECHO) conducted as an outpatient procedure in Germany, the United Kingdom, and Switzerland were associated with 50%, 25%, and 23% lower costs with the CMR-driven strategy, respectively. In contrast, when all tests were conducted in an outpatient setting in the United States, the CMR strategy was 8% more costly. The study concluded that the use of CMR could be a useful diagnostic tool to screen for myocardial ischemia for patients with suspected CAD and lead to better resource allocation for healthcare institutions outside of the United States [31]. In addition, some of the alternative options to screen myocardial viability such as PET require further testing whereas CMR results are conclusive prompting better treatment and cost saving through decreased testing [13].

### 4.6. The Role of Pretest Probability

One combination of imaging strategies investigated was CMR accompanied by invasive coronary angiography, which was compared with coronary angiography followed by FFR in the diagnostic workup of CAD. Although both coronary angiography and FFR were cost-effective for varying prevalence in some countries, CMR and coronary angiography combination was the most effective strategy across most countries [21]. Specifically, a lower prevalence of CAD was associated with higher cost-effectiveness for the CMR and coronary angiography strategy [21]. Higher cost-effectiveness was noted below the CAD prevalence of 62%, 65%, 83%, and 82% for Switzerland, Germany, United Kingdom, and United States healthcare systems, respectively [21]. In contrast, the coronary angiography and FFR strategy showed increasing cost-effectiveness in higher prevalence of CAD. Another study comparing the same three diagnostic pathways for CAD had similar supporting results [23]. In 3647 patients with suspected CAD, CMR plus coronary angiography minimized costs compared to coronary angiography with or without FFR [23]. In patients with typical angina, costs were reduced by 11.6 to 12.8% in the United States and Switzerland, respectively, and by 18.9% in the United Kingdom while having minimal savings in Germany (2.3%). The pretest probability, however, still guided the cost-effectiveness as cost savings in all four countries were higher for low pretest probabilities and decreased as pretest probabilities increased [23]. Both studies highlight the higher cost-effectiveness of CMR in low prevalence populations for the diagnosis of CAD by assessing the degree of cardiac ischemia and determining eligibility for revascularization.

A study in Germany comparing CMR, SPECT, and coronary angiography determined an inverse exponential curve between cost per diagnosis of CAD and prevalence of CAD for SPECT, angiography, and CMR [22]. The relation of this curve occurs because CMR and SPECT were able to rule out CAD at low prevalence rates, but with higher prevalence rates of CAD, patients needed additional testing or interventions [22]. Consequently, there would be a smaller number of invasive angiograms needed at a lower prevalence, suggesting CMR and SPECT are more cost-effective at a lower prevalence of CAD. However, with additional interventions and testing needed at a higher prevalence and increased severity of CAD for CMR and SPECT, there would be increased costs due to complications, and increased mortality favoring invasive angiography with more cost-effective results [22]. The study determined that at a low prevalence of CAD (<60%), CMR was the most cost-effective strategy followed by SPECT, but at a high prevalence of CAD (>60%), angiography was determined to be the most cost-effective strategy [22].

### 4.7. Evidence against Cost-Effectiveness of CMR

A few studies have shown CMR was not a cost-effective imaging technique at lower-to-intermediate pretest probabilities. One study conducted on 60-year-old patients with a low-to-intermediate pretest probability of CAD using a microsimulation model measured lifetime costs, QALYs, and incremental cost-effectiveness ratios (ICERs) to evaluate the most cost-effective imaging strategy in stable chest pain [32]. The study indicated that with low-to-intermediate probability of CAD, stress CMR and SPECT had less efficacy and were more expensive than stress ECHO. The study combined stress imaging with CT angiography and showed the best combination to be CT angiography with stress ECHO [32]. This study demonstrated that prevalence plays a significant role in determining the appropriate approach to a patient with CAD. Another study produced similar results when assessing the diagnosis of CAD in a European population with a low-to-intermediate prevalence of CAD from the Evaluation of Integrated Cardiac Imaging in Ischemic Heart Disease (EVINCI) study [33]. Cost-effectiveness analysis was performed in 350 patients with symptoms of CAD undergoing CCTA combined with one other cardiac imaging stress test, including stress ECHO, SPECT, PET, or stress CMR [33]. Effectiveness was defined as the percentage of correct diagnosis (cd) (i.e., obstructive CAD with >50% stenosis at quantitative coronary angiography in at least one major coronary vessel), and costs were calculated using country-specific reimbursements. Strategies combining stress CMR followed by CCTA or CCTA followed by stress ECHO, SPECT, or PET were all cost-effective [33]. ICERs were calculated using “no imaging” as a reference and indicated cost savings of −969 €/cd for CMR-CCTA, −1490 €/cd for CCTA-PET, −3092€ for CCTA-SPECT, and −3776 €/cd for CCTA-ECHO [33]. This study confirmed that there is no self-standing non-invasive imaging modality that is superior, but rather, the combination of CCTA with stress imaging is a cost-effective means to diagnose CAD and identify revascularization candidates prior to coronary angiography.

### 4.8. Pretest Probability in the United States

There has been limited available data from studies done in the United States regarding the use of CMR and cost-effectiveness analysis [34], besides contributions from the heterogeneity of payment and reimbursement systems in the United States. Notably, the Stress CMR Perfusion Imaging in the United States (SPINS) study was a multicenter cohort study that assessed the prognostic values of stress CMR and the associated costs of care in patients initially presenting with chest pain syndromes [44]. Utilizing data from the SPINS registry to assess the use of CMR as compared to SPECT, coronary angiography, and CCTA might be one of the first in its nature conducted in the United States [34]. SPECT has thus far been one of the principal non-invasive imaging methods performed prior to invasive angiography in the United States [34]. The results of this study suggest that prior to utilizing coronary angiography, CMR may be a better cost-effective modality for obstructive CAD [34]. In patients with a 32.4% probability of obstructive CAD, a CMR-based assessment of CAD was considered the best approach based on the $100,000/QALYs threshold in the United States [34]. These results were applicable to a wide range of patient populations adjusted for age, rate of revascularization, and cardiac events with different comorbidities. However, these results are applicable to the patient population presenting with stable chest pain syndromes with intermediate CAD prevalence of under 60%, beyond which coronary angiography becomes the preferred imaging modality. This further endorses that disease prevalence plays a large role in determining the cost-effectiveness of CMR.

### 4.9. Relationship with Morise Scores

Another investigation focused on the use of CMR preceding the need for invasive procedures [35]. This German study included 218 participants who were matched to a comparison group of the same size using age, gender, body mass index, diabetes, hypertension (HTN), and dyslipidemia. Furthermore, the study utilized relative value units (RVUs) to determine cardiac catheterization costs in Germany and CMS reimbursement to determine the costs of CMR. As the study reported that CMR was not reimbursed in Germany at the time, the cost was estimated using the ratio of CMR to catheterization with CMS data. These costs were compared with each other at different pretest probabilities for CAD and Morise scores, which is a validated score that stratifies for CAD by accounting for age, sex, symptoms, estrogen status, diabetes, and other pertinent pieces of patient history [45]. The two groups did not significantly differ in CAD risk factors and Morise scores, and the investigation found that CMR was associated with net savings of £90 per patient, being inversely correlated to the Morise score. Those with lower pre-test probability and Morise scores for CAD had higher catheterization avoidance rates [35]. The study also concluded that the lowest Morise score was associated with the highest cost savings indicating that CMR is more cost-effective for mild cases of CMR with better myocardial viability while catheterization is more cost-effective for more severe cases, as there is a higher chance that revascularization will be necessary.

## 5. Limitations of Cost-Effectiveness Analysis in CMR

As with other imaging modalities, CMR has its indications based on pathology, and its use varies regionally. One study compared the use of ECHO, cardiac CT, CMR, and nuclear imaging between the United States and England based on clinical practice guidelines and reimbursement schemes from Medicare or National Health Service (NHS) insurance [46]. It analyzed the imaging modalities over the 5-year range from 2011 to 2016 and discovered that the activity of CMR has increased over the years with the United States having 62 cases per 100,000 and England having 116 cases per 100,000 in 2016. The results suggested that CMR use has increased in the United States and England by a factor of 1.29 and 2.4, respectively, in the 5-year period [46]. The volume of imaging performed among different modalities is constantly increasing, which should be accounted for while making future predictions about cost-effectiveness.

There is significant heterogeneity within the United States regarding the use and payment methods for CMR. A retrospective analysis of 2012–2017 Part B Medicare physician payments from the Provider Utilization and Payment Data Physician and Other Supplier Public Use Files assessed the number of providers, locations, and physician reimbursements for CMR and cardiovascular CT [47]. In 2017, 582 physicians provided CMR services in 45 states, which is an 84.8% increase from 2012 [47]. Cardiovascular CT is more prevalent with services provided by 1645 physicians in 49 states, which is a 77.3% increase from 2012. Over the 6-year period, cardiovascular CT use increased by 97.4% while CMR use increased by 75.5% [46]. The use and payments for both CMR and CT vary widely by state, provider specialty, and setting, namely hospital vs. outpatient facility. In 2017, New York, Tennessee, Minnesota, Illinois, and Pennsylvania performed the highest number of CMR examinations [47]. Radiologists and cardiologists most commonly provided CMR services and received payments for them, but nationwide, only 1.0% of radiologists and 0.2% of cardiologists provided CMR services. Professional fees in the hospital setting increased over the study period while fees for the outpatient setting decreased due to changes in the billing code classification [47].

Finally, there is a constant flux of changes in the examination protocols, guidelines, and indications for different examinations, with CMR indications being added in every guideline revision. The clinical practice guidelines from the AHA change every year for CMR recommendations and there have been 12 updates between 2008 and 2018 [46]. These changes affect the comparisons and assumptions made for most cost-effectiveness methods.

## 6. Use of Artificial Intelligence in Cost-Effectiveness Analysis of CMR

Artificial intelligence (AI) has been a rapidly growing field in medical imaging where it is not only helping improve diagnostics [48] but has also found a rapidly growing role in patient workflow, decision-support tools, and clinical patient management in radiology departments [49,50,51]. AI in cardiovascular imaging has also been demonstrated to decrease the direct and indirect costs related to medical imaging ranging from imaging order and protocoling to image acquisition, interpretation, and evidence-based imaging recommendations [52]. However, not much has yet been investigated regarding the potential applications of AI and machine learning (ML) in the investigation of the cost-effectiveness of different cardiac imaging modalities. Future investigators are encouraged to further investigate the potential applications of AI and ML in the cost-effectiveness analysis of cardiovascular imaging.

## 7. Future Directions and Protocol Recommendations

Due to great variability in results discussed on the applicability of CMR, it can be difficult to find one definitive guideline on the recommended utilization of this imaging modality for any specific disease. Clinicians should remain aware of the overarching themes as they pertain to the cost-effectiveness and therapeutic utility of CMR. With relatively high prevalence of risk factors for CAD in the United States and European countries, there is likely a noticeable cost reduction with the use of non-invasive testing before invasive imaging in assessing acute coronary syndromes. Even though CMR has shown limited utilization across many healthcare institutions, one possible protocol in patients with stable CAD can be a stepwise imaging method that utilizes exercise stress testing and CMR followed by coronary angiography in patients with a low-to-intermediate pretest probability. This can be considered in conjunction with appropriate region-specific imaging guidelines and individualized Morise score calculations. Furthermore, because of high diagnostic accuracy, clinicians should be aware of the available literature that suggest a change in treatment optimization and patient diagnoses following the use of CMR. At this time, more data analyses are needed, similar to the SPINS registry, to make conclusive guideline recommendations in the United States.

## 8. Conclusions

With an increasing number of investigations elucidating its cost-effectiveness, CMR has great potential to be utilized as a standardized imaging modality to confirm cardiac conditions of ischemia/infarction, myocarditis, and infiltrative cardiac disorders. Given its increased sensitivity and specificity for CAD assessment and many other cardiac pathologies, CMR has shown cost-effectiveness for these cardiac conditions depending on the regional differences in incidence and prevalence and pretest probability thresholds used [20,25,36]. Moreover, a stepwise combination of utilizing different imaging modalities with specific indications in lieu of ACC/AHA guidelines can sometimes yield more cost-effectiveness in the long run, with higher QALYs compared to a single imaging modality alone [13,20,28,29].

## Figures and Tables

**Table 1:** Summary of Major Cost-Effectiveness Studies for Cardiovascular Magnetic Resonance Imaging.

Author	*n*	Study Population	Study Conclusion
Hegde et al., 2017 [13]	361	Cohort that met the ACC and AHA criteria for CMR imaging	CMR is cost-effective when used in conjunction with AHA and ACC, with overall net savings of $833,037 and per patient cost saving of $2308
Francis et al., 2013 [20]	11,040	Cohort of cardiology referrals in a European multicenter registry	Two-thirds of the patients experienced a change in therapeutic management and a minority (16%) had uncovered a new diagnosis
Moschetti et al., 2014 [21]	N/A	A cohort of CAD-positive patients	CMR followed by coronary angiography was the most cost-effective strategy across most countries, with a lower prevalence of CAD associated with a higher cost-effectiveness *
Boldt et al., 2013 [22]	N/A	Patient cohort with suspected CAD	CMR is more cost-effective at low-to-intermediate pretest probability (<60%) for CAD, and coronary angiography is more cost-effective at higher pretest probabilities (>60%)
Moschetti et al., 2015 [23]	3647	Patient cohort with suspected CAD from the European CMR registry	In patients with typical angina, CMR followed by coronary angiography strategy resulted in 2.3%, 11.6%, 12.8%, and 18.9% cost savings in Germany, United States, Switzerland, and United Kingdom, respectively *
Murphy et al., 2021 [24]	2000	Modeled population of patients with acute STEMI	Within one-year, routine CMR use increased spending by 14% per patient. Within seven years, CMR-guided management reduced costs by 3%
Stokes et al., 2019 [25]	N/A	Two models of multivessel CAD and unobstructive coronary arteries undergoing percutaneous coronary intervention	With CMR being introduced for all patient subgroups that utilize CMR, diagnostic accuracy may be a driver of its cost-effectiveness
Petrov et al., 2015 [26]	1158	Cohort of stable CAD patients	Patients undergoing CMR had a cost-savings of 12,466€ in hospital costs per life year
Pontone et al., 2016 [27]	600	Symptomatic CAD patients with a history of revascularization	Stress-CMR had a higher cost-effectiveness compared to CCTA
Pletscher et al., 2016 [28]	N/A	Patient cohort with stable angina from Switzerland with a prevalence rate of CAD of 39% and a base-case scenario of 60-year-old male patients	The most cost-effective strategy was exercise stress test, followed by CMR, then coronary angiography *
Walker et al., 2013 [29]	N/A	Cohort from the CE-MARC trial referred to cardiologists due to suspicion of angina pectoris	At a lower cost-effectiveness threshold of £20,000, the best diagnostic strategy was exercise stress test, then CMR, then coronary angiography. At a higher cost-effectiveness threshold of £30,000, the best strategy was CMR then coronary angiography *
Kozor et al., 2021 [30]	N/A	Cohort from the CE-MARC trial applied to the Austrian healthcare system	On a cost-effectiveness threshold of $45,000 to $75,000 QALYs, the most effective strategy was electrocardiogram stress testing, then CMR, then coronary angiography *
Moschetti et al., 2012 [31]	2717	A cohort of CAD-positive patients	When using inpatient coronary angiography, CMR strategy had 53.5% lower costs. All tests conducted outpatient in Germany, United Kingdom, and Switzerland were associated with a cost reduction of 50%, 25%, and 23% using the CMR-driven strategy, respectively *
Genders et al., 2015 [32]	N/A	60-year-old patients with low-to-intermediate pretest probability for CAD using a microsimulation model	At low-to-intermediate probability of CAD, stress CMR and SPECT had less efficacy and were more expensive than ECHO ^1^
Lorenzoni et al., 2019 [33]	350	European cohort with low-to-intermediate probability of CAD from the EVINCI study	ICER calculated using the CCTA-ECHO strategy yielded −3776€/correct diagnosis, in comparison to −969€/correct diagnosis *^1^
Ge et al., 2020 [34]	N/A	Patient cohort from the multicenter cohort SPINS registry	CMR-based assessment of CAD is the most cost-effective approach based on $100,000/QALY applicable to patients with a pretest probability of <60%
Pilz et al., 2011 [35]	218	German cohort with suspected CAD	Patients with lower pretest probability and Morise scores for CAD had higher catheterization avoidance rates and cost-savings with the CMR-driven strategy

* Stepwise diagnostic strategies are proceeded by additional imaging modalities if the prior ones are inconclusive or positive. ^1^ These studies provided evidence against the cost-effectiveness of CMR. (Abbreviations: STEMI—ST-Elevation Myocardial Infarction, CMR—Cardiac MRI, CAD—Cardiac Artery Disease, ACC—American College of Cardiology, AHA—American Heart Association, CCTA—Coronary CT Angiography, CE-MARC—Cardiovascular Magnetic Resonance and Single-Photon Emission Computed Tomography for Diagnosis of Coronary Heart Disease, QALY—Quality-adjusted life year, SPECT—Single-Photon Emission Computed Tomography, ECHO—echocardiography, EVINCI—Evaluation of Integrated Cardiac Imaging in Ischemic Heart Disease, ICER—Incremental Cost-Effectiveness Ratio, SPINS—Stress CMR Perfusion Imaging in the United States).

## Data Availability

Not applicable.
